# Ameliorating Drought Effects in Wheat Using an Exclusive or Co-Applied Rhizobacteria and ZnO Nanoparticles

**DOI:** 10.3390/biology11111564

**Published:** 2022-10-25

**Authors:** Faqeer Muhammad, Muhammad Aown Sammar Raza, Rashid Iqbal, Faisal Zulfiqar, Muhammad Usman Aslam, Jean Wan Hong Yong, Muhammad Ahsan Altaf, Bilal Zulfiqar, Jawad Amin, Muhammad Arif Ibrahim

**Affiliations:** 1 Department of Agronomy, Faculty of Agriculture and Environment, The Islamia University of Bahawalpur, Bahawalpur 63100, Pakistan; 2 Department of Horticultural Sciences, Faculty of Agriculture and Environment, The Islamia University of Bahawalpur, Bahawalpur 63100, Pakistan; 3 Department of Biosystems and Technology, Swedish University of Agricultural Sciences, 23456 Alnarp, Sweden; 4 School of Horticulture, Hainan University, Haikou 570228, China

**Keywords:** plant growth promoting rhizobacteria, nanoparticles, *Azospirillum*, drought, biostimulant, crop resilience, antioxidants

## Abstract

**Simple Summary:**

Wheat is a vital source of food, and its production is increasingly threatened by drought episodes. Moreover, its cultivation under water deficit situations along with zinc deficient soils is a major concern of declined wheat grain quantity and quality. Drought-linked changes in nutrient use efficiency, photosynthetic mechanisms, and chemical composition of wheat plants ultimately led to poorer harvest. Therefore, we aimed to understand the drought-ameliorating and grain nutritional improving effects in wheat by rhizobacteria (*Azospirillum brasilense*) and ZnO nanoparticles (NPs) under various growth stage-based drought stress episodes. Rhizobacteria colonized the host plant rhizosphere and provided growth promotion and stress amelioration. ZnO NPs were recognized as a potential water and zinc deficiency alleviator, and general growth promoter by triggering nitrogen metabolism, chlorophyll synthesis, membrane integrity, and grain zinc biofortification activities. Physio-biochemical observations indicated significantly higher positive effects under co-application over the sole use of either microbial or nanomaterials. Based on our research, it was concluded that co-applied *Azospirillum brasilense* and ZnO NPs generally increase wheat productivity under drought episodes with low operational cost to growers. Further, plausible synergistic physiological enhancement by NPs and rhizobacteria interaction may contribute towards sustainable wheat crop management under abiotic stresses.

**Abstract:**

Drought is a major abiotic factor and affects cereal-based staple food production and reliability in developing countries such as Pakistan. To ensure a sustainable and consistent food supply, holistic production plans involving the integration of several drought mitigation approaches are required. Using a randomized complete block design strategy, we examined the drought-ameliorating characteristics of plant growth-promoting rhizobacteria (PGPR) and nanoparticles (NPs) exclusively or as a combined application (T_4_) through three stages (D_1_, D_2_, and D_3_) of wheat growth (T_1_, control). Our field research revealed that *Azospirillum brasilense* alone (T_2_) and zinc oxide NPs (T_3_) improved wheat plant water relations, chlorophyll, proline, phenolics and grain quality, yield, and their allied traits over the stressed treatments. Specifically, the best outcome was observed in the combined treatment of PGPR and ZnO NPs (T_4_). Interestingly, the combined treatment delivered effective drought mitigation through enhanced levels of antioxidants (15% APX, 27% POD, 35% CAT, 38% PPO and 44% SOD) over controls at the grain-filling stage (GFS, D_3_ × T_1_). The 40% improvements were recorded under the combined treatment at GFS over their respective controls. Their combined usage (PGPR and ZnO NPs) was concluded as an effective strategy for building wheat resilience under drought, especially in arid and semi-arid localities.

## 1. Introduction

Drought is a major and unavoidable abiotic stress targeting numerous regions across the globe by subsisting in various environments without recognizing borders and with no clear warning. Therefore, it may hamper intensively the plant production, plant protection, and quality of the produce under the influence of its causative factors such as temperature crescendos, light intensity, and low rainfall [1]. Further, Shahzad et al. [2] ranked it as the topmost environmental stressor affecting all factors related to safeguarding cereal food security from production to post-harvest consumption. Drought is considered a dominant factor causing poor wheat productivity [3] because of its adverse effects on critical growth stages [4]. Gull et al. [5] recounted the belligerent impacts of drought on different junctures of growth in wheat. Its occurrence at tillering may result in low plant height and total tillers per unit area, followed by less biomass, spiked tillers, grains per spike, and finally, low grain weight at the grain-filling stage, which may cause 50% yield losses [6,7,8].

Tambussi et al. [9] documented that the highly reactive oxygen species (ROS) under drought in wheat is a leading cause of gradual degradation of its photosynthetic system. Furthermore, ROS consists of H_2_O_2_, superoxide, and hydroxyl radicals, and all of these finally lead to cell death through augmented DNA denaturation and protein deprivation [10,11]. Bakht et al. [12] highlighted various adaptations to alleviate drought at cellular and sub-cellular levels. Intensity and duration of drought exposure further alter plant physiology and morphology [13], tending to avoid water loss by root, leaf, and stem adaptations, production, and mobilization of stress proteins, antioxidants, and metabolites [14]. The antioxidant security system is a self-safeguarding mechanism to avoid ROS damage in plants. Antioxidant enzymes such as Ascorbate peroxidase (APX) and Catalase (CAT) mostly detoxify H_2_O_2_ in chloroplasts and mitochondria, respectively [15,16]. Polyphenol oxidase (PPO) further lessens oxidative injury in wheat plants at the grain-filling stage [17].

Mishra et al. [18] discussed the natural and human disasters of the 21st century and ascribed the disrupted global food supplies to many drought episodes and COVID-19 pandemics. They further elaborated that the compound effects of natural (drought) and human (COVID-19) disasters could create an austere fiscal loss besides severe starvation. Hence, to avoid the consequences of such disasters, food scientists are reframing plausible defensible solutions that may improve plant production potentials under limited natural water resources [19]. The nutrients are crucial for plant development, and the use of nanoparticles as a source of micro and macro nutrients could further enhance plant productivity and stress tolerance [20]. Freitas et al. [21] reviewed that there is room for more research on the effects of nanoparticles in mediating plant stress responses for future agricultural research developments. Interestingly, nanoparticles (NPs) were found to be fueling plant growth by facilitating efficient input delivery networks and enriching plant health [22]. Zinc oxide nanoparticles (ZnO NPs) are known to alter the levels of Fe, Zn, Mn, P, S, Mg, K, glutelin, and globulin through soil application in cucumber and pea plants [23,24]. Similarly, improved germination, foliar chlorophyll, and yield in groundnuts and maize were reported by Prasad et al. [25] and Subbaiah et al. [26] upon application of ZnO NPs at 1000 and 100 mg L^−1^, respectively.

In recent years, advancements in biochemical and molecular tools significantly contributed to unveiling the mode of actions of microbes in mediating plant growth and resilience to stress [27,28]. Zhang et al. [29] appraised that the plant growth promoting rhizobacteria (PGPR) are very important microbes to sustain plant growth and to minimize the adverse effects of drought. Kumar et al. [30], while discussing the various biological tools available to mitigate salinity stress, conveyed that the *Azospirillum brasilense* inoculation could improve the drought resilience, growth, and biological yield in wheat. Likewise, *Azospirillum brasilense* was also concluded as a plausible experimental approach by Aslam et al. [31] while alleviating drought stress in quinoa crops.

Urban farming, besides conventional farming, is an innovative remedy to address the major challenges of today’s world, including the climate change-mediated scarcity of natural resources (water resources). It provides more eco-sustainable food distribution along with improved carbon footprint and avoiding transportational food wastage from rural areas to big cities. The use of beneficial soil microbiota (microorganisms–PGPR) and nanomaterials (NPs) were also found as useful “tools” to accelerate the evolution of urban farming due to their ability to facilitate controlled release of nutrients, optimized water retention capability and better plant health in both indoor and outdoor cultivation scenarios [32].

The amalgamation of drought coping strategies instead of their alone use may boost agricultural yields [28,33]. In this context, a combination of ZnO NPs with PGPR holds great promise in the agriculture sector [34]. Bhatia et al. [35] regarded their co-application protocol as a plausible alternative for sustainable crop management. With new research efforts, the various PGPR and NPs combinations are known to improve the effectiveness of NPs and ameliorating the nanotoxicity-related health hazards [22,36]. Successful experimentation of PGPR and ZnO NPs had been reported in wheat and *Cucumis melo* under salinity and heavy metal stresses, respectively [37,38].

The current study examined the effects of applying *Azospirillum brasilense* and zinc oxide NPs under field conditions at tillering and grain-filling stages of wheat undergoing drought episodes. We hypothesized that the combined application of PGPR and ZnO NPs would improve the physiology, productivity, and grain quality of wheat more efficiently during drought as compared to their individual usage.

## 2. Materials and Methods

### 2.1. Experimental Layout and Crop Husbandry

A field investigation was conducted during 2020–2021 at the agronomic research area, Faculty of agriculture and environment, The Islamia University of Bahawalpur, Pakistan. The soil profile of experimental site included Texture Class = loam soil, and the other soil physiognomies enclosed; pH = 7.98, EC = 242 (µS cm^−1^), soil available P and K = 8.68:119 (ppm), Total N = 0.112, Zinc = 0.945, and sand, silt and clay (% age) = 38:40:22. The weather specifications of the location are as follows: rainfall = 15.50 mm and temperature = 28.17 °C on average during growing season. Seeds of wheat genotype (Galaxy 2013) were inoculated with rhizobacteria (*Azospirillum brasilense*), and un-inoculated were considered as control. The Galaxy 2013 genotype of wheat is known to grow well in the Bahawalpur district (semi-arid region) due to its good yield potential and the superior baking quality of its flour. Drought was applied at tillering (TS) and grain-filling stages (GFS), while the plants receiving normal irrigation were considered as the “no drought” (control) treatment. The zinc oxide NP (150 mg L^−1^) solutions were applied through irrigation under normal conditions as well as when drought was planned at particular growth stages. The experiment was arranged according to randomized complete block design (RCBD) in factorial settings, having three replications under the following experimental factorization; **Factor 1.** Three drought levels: D_1_ = no drought (control), D_2_ = drought at tillering stage (DTS), and D_3_ = drought at grain-filling stage (DGS). **Factor 2.** Four treatments: T_1_ = control, T_2_ = sole usage of *Azospirillum brasilense* (PGPR), T_3_ = sole usage of zinc oxide nanoparticles (ZnO NPs), and T_4_ = co-applied PGPR + ZnO NPs.

The PGPR inoculated and un-inoculated wheat seeds were sown in different plots with a single coulter hand drill @ 22 cm Row × Row spacing with 3 × 5 m plot dimensions in November 2020. Recommended fertilization and irrigation scheduling was followed. The traditional irrigation protocol (three acre inches irrigation per plot) was applied except during the tillering and grain-filling stages and when scheduled drought stress was planned. Further, the crop was provided with 1st irrigation after 21 days of sowing and, later on, was irrigated as per plant and plot requirements except for drought-induced plots at specified growth stages. Fertilizer was applied using 120 kg N and 80 kg P_2_O_5_ ha^−1^ in each plot. The entire phosphorus and 1/2 nitrogen were applied as basal dose, and the remaining was applied at the flowering stage. In the fields, three feet (91.4 cm) inter-plot distancing was maintained to avoid flooding from adjacent plots.

### 2.2. Drought Imposition

All the plots (area of each plot = 15 m^2^) received the same irrigation until the imposition of the simulated drought episode. The experimental units (plots) receiving unrestricted irrigation throughout the growth period were designated as having no drought (D_1_). The drought was imposed by halting the irrigation at tillering and grain-filling stages and labeled as D_2_ (DTS) and D_3_ (DGS) drought levels, respectively.

### 2.3. PGPR and Nanoparticles Application

The PGPR strain *Azospirillum brasilense* and wheat variety Galaxy 2013 were collected from government research institutes located at Bahawalpur and Faisalabad—PK (Regional agriculture research institute—RARI, Ayub agricultural research institute—AARI), respectively. ZnO NPs (<100 nm) were purchased from Sigma Aldrich, USA. The seed surface sterilization with 70% ethanol (1 min) and with 3% sodium hypochlorite (NaOCl) for 30 min was conducted, and then, the seeds were washed with sterilized water five times. Afterward, these seeds were inoculated with 10^8^ colonies forming units per milliliter, attained at the exponential microbial growth phase of PGPR (10^8^ CFU ml^−1^, *Azospirillum brasilense*), further adjusted at 150 cm^3^/50 kg seeds for field trial. ZnO NPs were soil applied at 150 mg L^−1^ in each respective experimental unit according to the layout plan. The individual and combined treatments of PGPR and ZnO NPs were carried out accordingly (T_2_ = alone PGPR, T_3_ = alone ZnO NPs, T_4_ = combination of PGPR and ZnO NPs), and the treatments without PGPR and ZnO NPs application were termed as control (T_1_).

### 2.4. Data Recording and Related Procedures

#### 2.4.1. Growth and Yield Parameters

The tillers were counted per square meter. Nodes per stem were counted by using these tillers. The inter-nodal length was recorded by measuring tape. Plant height was taken from ground level to the terminal node of the spike. The spike length of randomly selected 25 plants was measured with measuring tape. Grains were totaled by threshing these spikes separately and weighed on a digital balance. Two intermediate rows were harvested to record biological and grain yields in every plot. The proportion of grain yield (economic returns) to total biological harvest was used for harvest index calculation [39].

#### 2.4.2. Wheat Physiological Characteristics

Following the practice by Khalilzadeh et al. [40], 0.5 g of fresh leaf tissue was gradually added with 10 mL acetone (80%) to measure chlorophyll contents. It was centrifuged at 400 rpm for 10 min, and the absorbance at 645 nm, 663 nm, and 470 nm was recorded by a spectrophotometer. The chlorophyll and carotenoid contents were obtained as follows:

$$\mathrm{Chlorophyll}\;a=\left(19.30\times A663-0.86\times A645\right)\times \left(V/100W\right)$$

$$\mathrm{Chlorophyll}\;b=\left(19.30\times A645-3.6\times A663\right)\times (V/100\;W)$$

Total Chlorophyll=Chlorophyll a+Chlorophyll b

$$\mathrm{Carotenoids}=\left(1000\;A470-1.82\;\mathrm{Chl}.a+85.02\;\mathrm{Chl}.b\right)$$

The transpiration rate was measured using a MK-3 Porometer (Delta–T Dev., Burwell –Cambridge, Herts, England).

RWC (leaves) were calculated as follows [41]:

$$\mathrm{RWC}=\frac{W_{f}-W_{d}}{W_{t}-W_{d}}\times 100$$

Leaf water potentials were measured using the second leaf (from the top) through a gadget (600 L model, C.W. Cook, and Sons Ltd., England) for each treatment. Leaf osmotic potential was determined through an Osmometer (Wescor-5520, Logan, UT, USA).

#### 2.4.3. Quality Parameters

The foliar elemental (N, P, K, Zn, and Fe) concentrations were measured in accordance with Wolf [42]. Grain quality traits such as protein and zinc concentrations were also analyzed. Prior estimated grain N content of grain digested material was converted into protein content by the given formula [43]: N content × 5.70 = Protein content. Subsequently, by using Inductively coupled plasma atomic emission spectroscopy (ICP-OES, Optima2100—DV, Perkin-Elmer, Waltham, MA, USA), zinc concentration was determined in digested grain samples.

#### 2.4.4. Enzymatic and Non-Enzymatic Antioxidants Activity Assays

We measured CAT, POD, and PPO activities using the procedure reported by Kar and Mishra [44]. The activity of APX (Ascorbate peroxidase) was measured as per Cakmak [45]. The determination of leaf proline and phenolic contents was conducted in accordance with standard methods designated by Bates et al. [46] and Waterhouse [47], respectively.

### 2.5. Data Processing Software Usage for Analysis

Analytical Software, Saint Paul, MN (Statistix 8.1; computer-installed software) was used to statistically analyze the data of all parameters. The divergences among the treatment means were compared by using ANOVA techniques and LSD (5% probability level).

## 3. Results

### 3.1. Growth and Yield Characters

Results in Table 1 and Table 2 revealed interesting effects of drought on growth parameters (PT, Internodal lengths, PH, SL, and NSPS) under the D_2_ level (Drought at tillering) while yield and yield components (grain numbers per spike, 1000 grain weight, grain yield, and HI) suffered the highest loss at grain-filling stage (D_3_, DGS). The D_1_ (No drought) treatment showed the highest results regarding all of these parameters. Application of *Azospirillum brasilense* (PGPR) (T_2_) and ZnO NPs (T_3_) improved these attributes by mitigating drought stress at critical growth stages as compared to control (T_1_—No PGPR or NPs) under respective drought levels. Interestingly, the highly significant results were achieved by co-applied PGPR and ZnO NPs (T_4_) over respective T_1_ at each drought level.

The highest reduction in productive tillers (24%), Int.L (10%), PH (56%), SL (33%), and spikelets (14%) were observed in T_1_ at tillering under drought over control × D_1_ (Table 1) while 20%, 39%, 43%, and 18% decreased number of grains, 1000 grain weight, grain yield, and harvest index were observed at grain-filling stage (D_3_ × T_1_), respectively, as compared to control (Table 2). A maximum percent increase in all the parameters was recorded by T_4_ (PGPR + NPs) at D_1_ (no drought). Moreover, T_4_ enhanced 7% and 10% more PT per meter square, 1% and 5% more internodal length, 14% and 18% more SL, 1.4% and 4% more PH, 0.7% and 13% more SY, 1.2% and 4% more 1000 grain weight, and 6% and 3% more GY at tillering stage under drought (D_2_) as compared to alone use of PGPR (T_2_) and ZnO NPs (T_3_) respectively. Similarly, 8.5% and 9.3% more PT, 0.7% and 3% more nodal space, 23.5% and 24.3% more SL, 2% and 3% more PH, 1.7% and 3.1% more SY, 7% and 14% more 1000 grain weight, and 24% and 28% more GY were achieved by T_4_ as compared to T_2_ and T_3_ respectively at water-stressed grain-filling stage (D_3_). Therefore, these results confirmed the negative effects of drought; on growth at tillering and on yield attributes at GFS, followed by restoration through T_4_ in these parameters as compared to T_2_ and T_3_.

### 3.2. Plant Morphological and Grain Quality 

The morphological and quality parameters were significantly improved by co-application of PGPR and NPs (T_4_) followed by a single use of PGPR (T_2_) and NPs (T_3_), respectively, as compared to the control (Table 3). The highest increase in NS^−1^ (34%) and grain weight per spike (17%) was achieved at D_3_ and D_2_, respectively, followed by 28% increased NS^−1^ and 13% (SpWt.) at D_1_ by T_4_ over respective controls (T_1_). Lowest NFT was recorded by application of T_4_ under non-drought conditions. Maximum grain protein (31%) and zinc (117%) concentrations were measured in D_1_ and D_3_, respectively, followed by 8% increased protein at D_3_ and 52% increased zinc at D_1_ through the application of T_4_ as compared to respective controls (T_1_).

### 3.3. Physiological Characteristics

**Figure 1:**
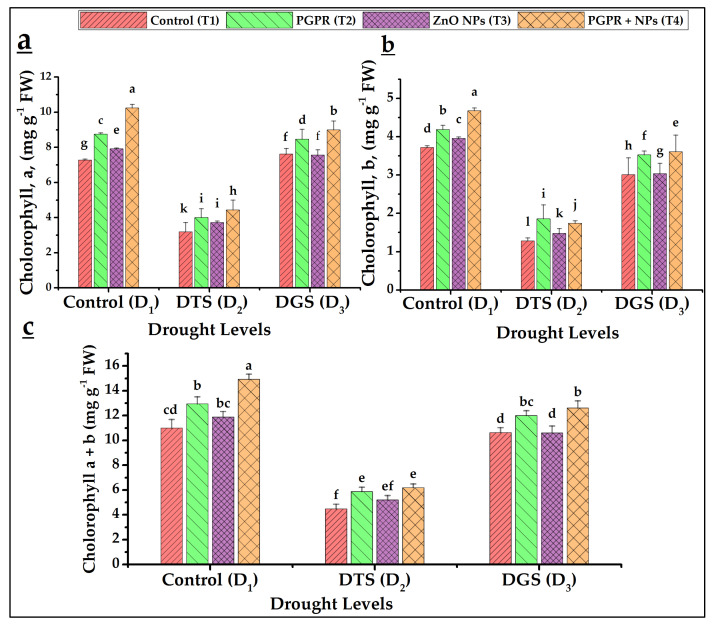
The effects of PGPR and NPs application on foliar chlorophyll contents on wheat under drought. (**a**) Chlorophyll a, (**b**) Chlorophyll b, (**c**) Total chlorophyll content. DTS = drought at tillering, DGS = drought at grain filling, T_1_ = control, T_2_ = single use of *Azospirillum brasilense*, T_3_ = single use of ZnO NPs, and T_4_ = co-application of PGPR and NPs. Error bars indicates standard error (*n* = 3). (Note: Different lowercase letters indicate significant differences between different drought levels and treatments by the LSD test at *p* < 0.05).

Figure 1a–c indicated reduced foliar chlorophyll contents under respective drought-stressed controls (T_1_) at D_2_ and D_3_ as compared to the D_1_ control. The application of PGPR and NPs had a significant effect on the chlorophyll contents of wheat leaves under drought stress. The best values of chl. a, b, and total chl. (10, 5, and 15 mg g^−1^ Fw, respectively) were observed using T_4_ at D_1_ followed by T_2_ over control. Under D_3_, the combined application of PGPR and NPs (T_4_) resulted in the highest chl.a (102%), chl.b (20%) and total chl. (18%) over respective controls and applications of T_2_ and T_3_ under different drought levels.

**Figure 2:**
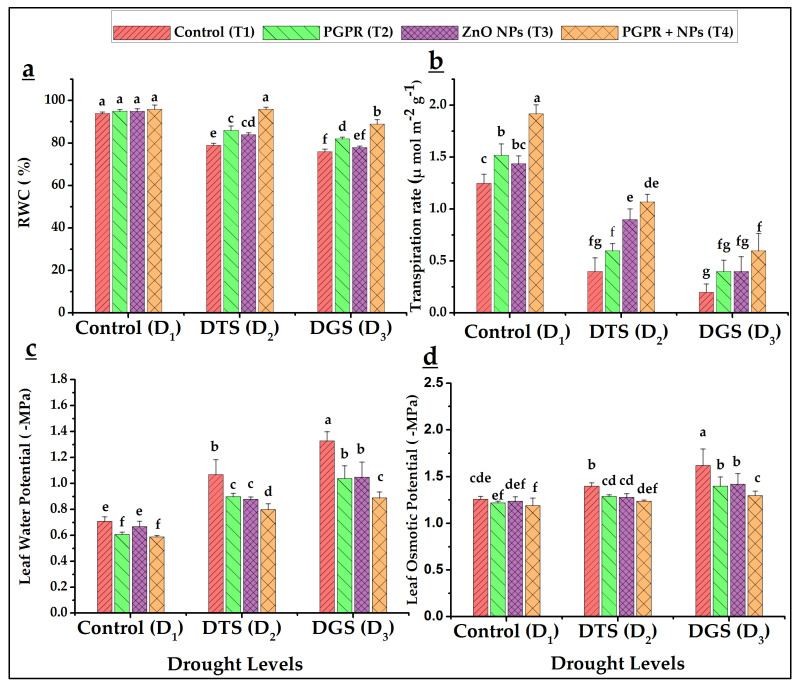
Effects on relative water content modifications RWC (**a**), transpirational rates (**b**), foliar water and osmotic potentials (**c**,**d**) in drought-subjected wheat by single and co-applied ZnO NPs and *Azospirillum brasilense* (PGPR). DTS = drought at tillering, DGS = drought at grain filling, T_1_ = control, T_2_ = single use of *Azospirillum brasilense*, T_3_ = single use of ZnO NPs, and T_4_ = co-application of PGPR and NPs. Error bars indicates standard error (*n* = 3). (Note: Different lowercase letters indicate significant differences between different drought levels and treatments by the LSD test at *p* < 0.05).

Foliar transpiration rates and relative water contents at D_1_ were highest in the treatment with the co-use of PGPR and NPs, followed by T_2_, T_3_, and T_1_ (Figure 2a,b). Drought stress decreased both parameters at D_2_ and D_3_. However, T_4_ significantly enhanced the transpiration rates and RWC under D_2_ and D_3_ over respective controls. T_4_ increased by 78% and 18% more transpiration as compared to T_2_ and T_3_ at D_2_ and up to 50% at D_3_ when compared with T_2_ and T_3_. In total, 21% and 17% enhanced relative water contents were observed when T_4_ was applied at D_2_ and D_3_, respectively, as compared to T_1_ at the same drought levels.

The LWP (Leaf water potential) and LOP (Leaf osmotic potential) displayed significant differences between growth-stage-based drought treatments (D_2_ and D_3_) and drought-ameliorating materials (NPs and PGPR). Drought reduced LWP and LOP (numerically high –MPa values) in affected wheat plants at D_3_ and D_2_, respectively. The ZnO NPs and *Azospirillum brasilense* (alone or combined) have the potential to restore LWP and LOP under water-stressed environments to similar levels as well-watered plants (Figure 2c,d), and 11% and 33% and 20% decreased LWP and LOP were achieved by T_4_ as compared to respective T_1_ at D_2_ and D_3_, respectively.

### 3.4. Nutrients Uptake Attributions

**Figure 3:**
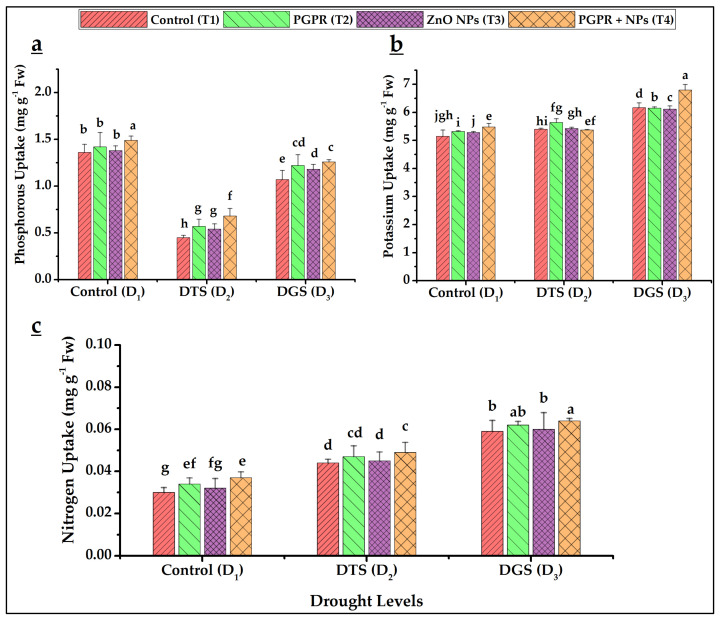
Phosphorus (**a**), potassium (**b**), and nitrogen (**c**) uptake in wheat as affected by *Azospirillum brasilense* and ZnO NPs under drought conditions. DTS = drought at tillering, DGS = drought at grain filling, T_1_ = control, T_2_ = single use of *Azospirillum brasilense*, T_3_ = single use of ZnO NPs, and T_4_ = co-application of PGPR and NPs. Error bars indicates standard error (*n* = 3). (Note: Different lowercase letters indicate significant differences between different drought levels and treatments by the LSD test at *p* < 0.05).

**Figure 4:**
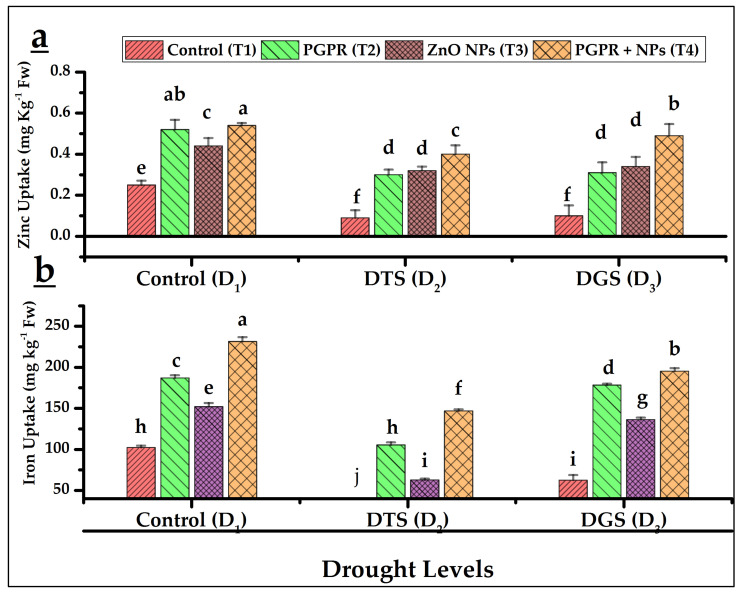
Effects of PGPR and ZnO NPs on zinc (**a**) and iron (**b**) uptake in wheat under different drought levels. DTS = drought at tillering, DGS = drought at grain filling, T_1_ = control, T_2_ = single use of *Azospirillum brasilense*, T_3_ = single use of ZnO NPs, and T_4_ = co-application of PGPR and NPs. Error bars indicates standard error (*n* = 3). (Note: Different lowercase letters indicate significant differences between different drought levels and treatments by the LSD test at *p* < 0.05).

The results regarding N, P, K, Zn, and Fe uptake by leaves revealed enhanced uptake of these nutrients occurred under respective drought-stressed controls (T_1_) of D_2_ and D_3_ as compared to T_1_ at D_1_ except iron, whose uptake was severely suppressed under stressful conditions in the absence of any drought ameliorating materials. However, the uptake of all these nutrients further improved under the co-application of PGPR and NPs at all drought levels. Maximum uptake of N (8%) and K (2%) at D_3_ while highest uptake of P (9%), Zn (116%), and Fe (126%) were achieved by T_4_ at D_1_ as compared to T_1_ of both drought levels (Figure 3a–c and Figure 4a,b).

### 3.5. Biochemical Characteristics

**Figure 5:**
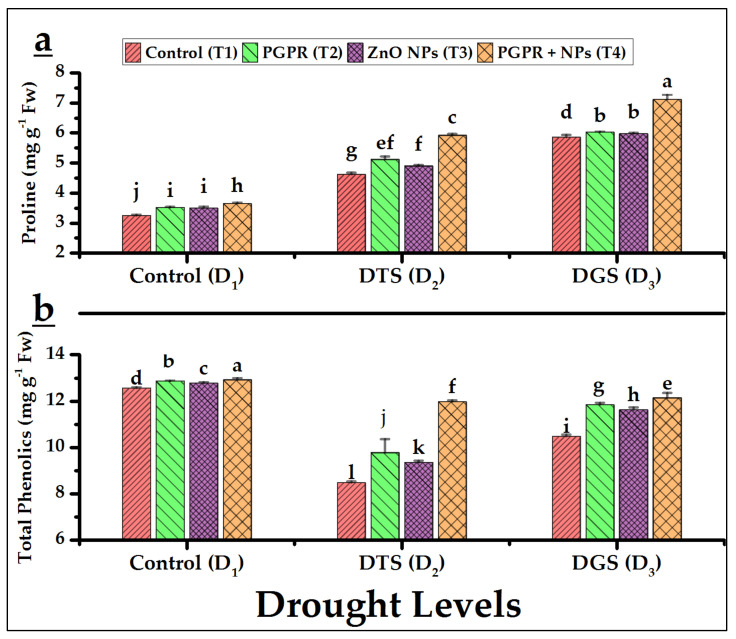
Effects of PGPR and ZnO NPs on foliar proline (**a**) and total phenolics (**b**) in drought-stressed wheat. DTS = drought at tillering, DGS = drought at grain filling, T_1_ = control, T_2_ = single use of *Azospirillum brasilense*, T_3_ = single use of ZnO NPs, and T_4_ = co-application of PGPR and NPs. Error bars indicates standard error (*n* = 3). (Note: Different lowercase letters indicate significant differences between different drought levels and treatments by the LSD test at *p* < 0.05).

The levels of proline and total phenolics under drought at tillering (D_2_) and grain-filling (D_3_) stage in wheat by the application of PGPR and NPs are shown in Figure 5a,b. Maximum proline and total phenolics (TP) were recorded with the T_4_ application. We observed a 21% and 15% increase in proline and TP levels under the co-application of PGPR and NPs (T_4_) at D_3_ as compared to its respective control.

**Figure 6:**
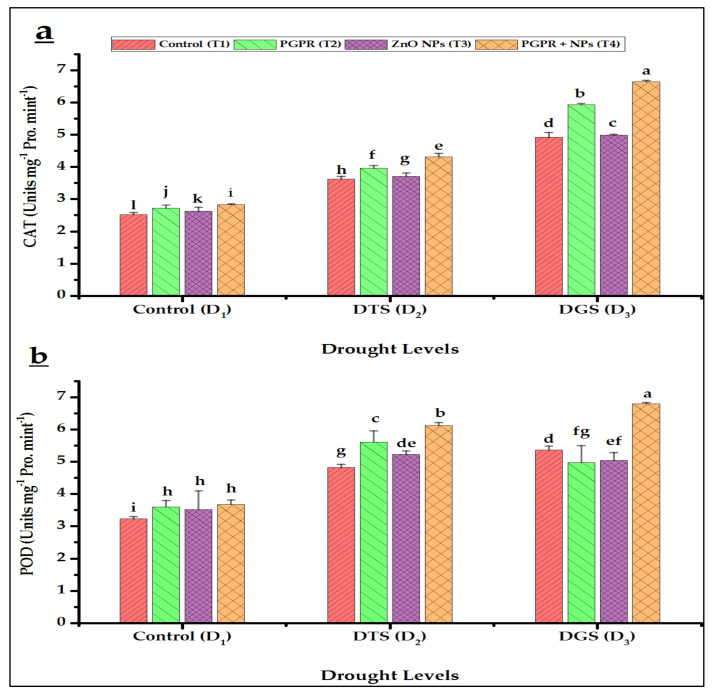
Effects of *Azospirillum brasilense* and ZnO NPs on wheat catalase (**a**) and peroxidase (**b**) activities under drought conditions. DTS = drought at tillering, DGS = drought at grain filling, T_1_ = control, T_2_ = single use of *Azospirillum brasilense*, T_3_ = single use of ZnO NPs, and T_4_ = co-application of PGPR and NPs. Error bars indicate standard error (*n* = 3). (Note: Different lowercase letters indicate significant differences between different drought levels and treatments by the LSD test at *p* < 0.05).

**Figure 7:**
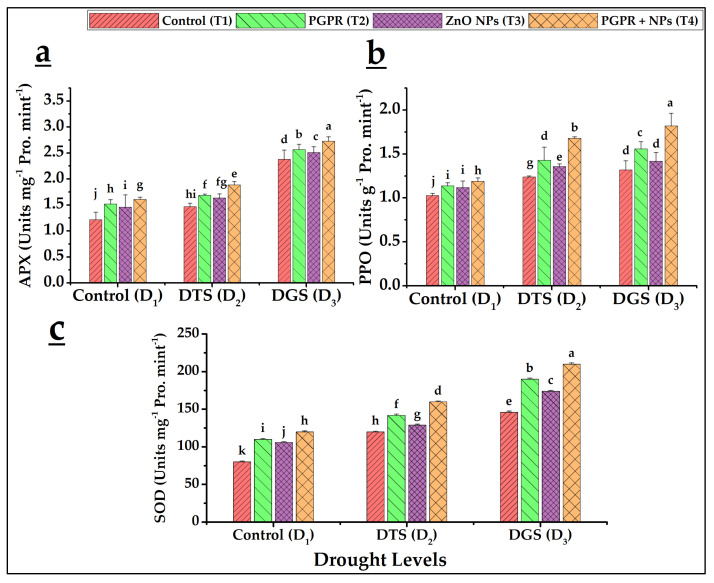
Effects of *Azospirillum brasilense* and ZnO NPs applications on ascorbate peroxidase (**a**), polyphenol oxidase (**b**), and superoxide dismutase (**c**) activities in wheat under drought. DTS = drought at tillering, DGS = drought at grain filling, T_1_ = control, T_2_ = single use of *Azospirillum brasilense*, T_3_ = single use of ZnO NPs, and T_4_ = co-application of PGPR and NPs. Error bars indicate standard error (*n* = 3). (Note: Different lowercase letters indicate significant differences between different drought levels and treatments by the LSD test at *p* < 0.05).

Our results indicated that increased activities of APX, PPO, CAT, POD, and SOD were observed for T_1_ under drought imposition at D_2_ and D_3_ as compared to T_1_ with no drought imposition (D_1_). The highest antioxidant enzyme activity was observed at GFS during the tillering of wheat (Figure 6 and Figure 7). The maximum values of antioxidants were found with the combined interaction of *A. brasilense* and ZnO NPs (T_4_) at D_3_ and D_2_, respectively. We observed 19% and 35% increased CAT and 35% and 26% increased POD activities for D_2_ and D_3_, respectively, by T_4_ over their T_1_ (Figure 6a,b). In addition, 28% and 14% APX and 35% and 37% increased PPO activities were recorded at D_2_ and D_3_, respectively, by the application of T_4_ as compared to their respective controls (T_1_) (Figure 7a,b). Similarly, SOD showed 33% and 44% enhanced performance at D_2_ and D_3_ by T_4_ in comparison with its T_1_ (Figure 7c).

### 3.6. Principal Component Analysis (PCA)

**Figure 8:**
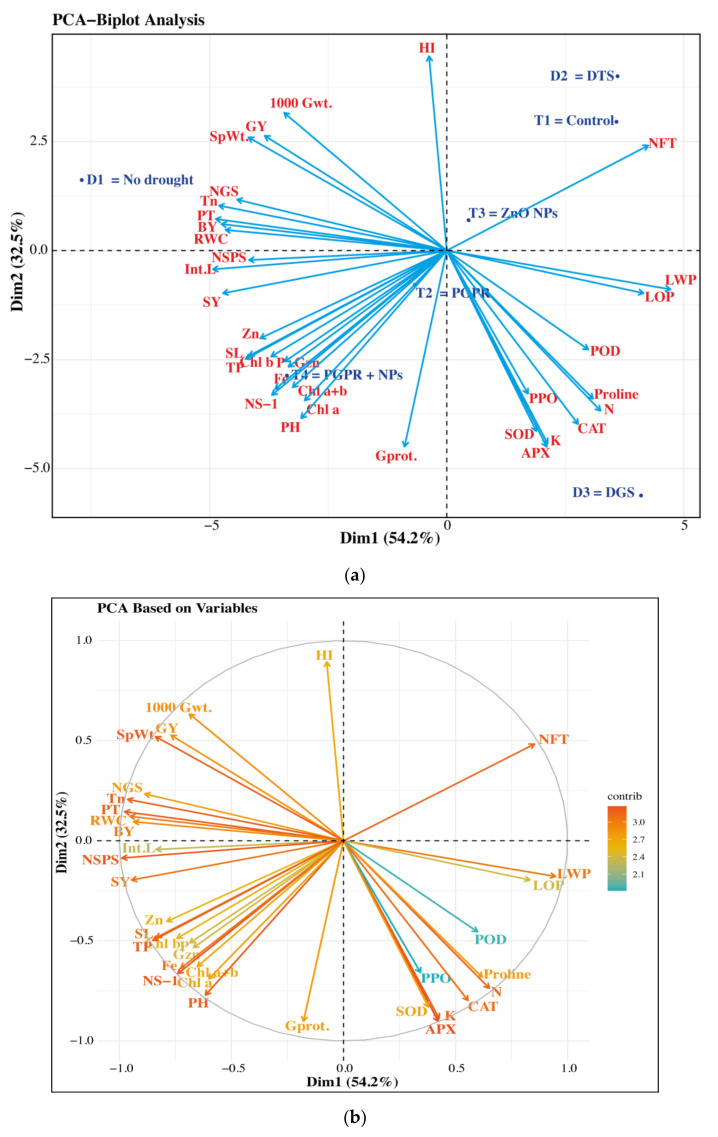
(**a**) The Principal Component Analysis (PCA) was based on different parameters of wheat under different treatments of *Azospirillum brasilense*, ZnO NPs, and drought stress. **(b)** The PCA was performed using variables with contribution of different parameters from wheat under PGPR, ZnO NPs, and drought stress. NFT = Non fertile tillers (m^−2^); LWP = Leaf water potential (MPa); LOP = Leaf osmotic potential (MPa); POD = Peroxidase (units mg^−1^ pro. mint^−1^); PPO = Polyphenol oxidase (units g^−1^ pro. mint^−1^); CAT = Catalase (units mg^−1^ pro. mint^−1^); SOD = Superoxide dismutase (units mg^−1^ pro. mint^−1^); APX = Ascorbate peroxidase (units mg^−1^ pro. mint^−1^); N, P, and K = Nitrogen, phosphorous and potassium foliar uptakes (mg g^−1^ FW); Zn and Fe = zinc and iron foliar uptakes (mg kg^−1^ FW); Gprot. = Grain protein (%); Gzn = Grain zinc content (mg Kg^−1^ DW); Chl. a, b and a + b = Chlorophyll a, b and a + b(mg g^−1^ FW); TP = Total phenolics (mg g^−1^ Fw); PH = Plant height (cm), NS^−1^ = Nodes per stem; SL = spike length (cm); Int.L = Inter-nodal length (cm); NSPS = Number of spikelets per spike; SY = Straw yield (T ha^−1^); RWC = Relative water contents (%); BY = Biological yield (T ha^−1^); PT = Number of productive tillers (m^−2^); NGS = Number of grains per spike; GY = Grain yield (T ha^−1^); SpWt. = Grain weight per spike (g); 1000 Gwt. = 1000 grain weight (g); HI = Harvest index (%); Tn = Transpiration rate (µmol m^−2^ s^−1^); Dim1 = First principal component; Dim2 = Second principal component; DTS = Drought at tillering stage; DGS = Drought at grain-filling stage; Contrib = Contribution.

A principal component analysis (PCA) was performed to analyze the variations and associations among different growth, physiological, morphological, biochemical, yield, and quality-linked attributes of wheat under the application of PGPR (*Azospirillum brasilense*), ZnO NPs and drought stress. The treatments and different parameters were scattered into different clades within the PCA—Biplot Analysis (Figure 8a). The first two principal components (PC1 and PC2) accounted for 86.7% variability of the total variations derived from the 35 parameters. The first principal component (PC1, 32.5%) was related to 11 parameters regarding growth, antioxidants, water relations, and quality of wheat plants, while the remaining 24 parameters were attributed to the second principal component (PC2, 54.2%). All the treatments (T_1_ = control, T_2_ = single use of *Azospirillum brasilense*, T_3_ = single use of ZnO NPs, and T_4_ = co-application of *A. brasilense* and ZnO NPs) under different drought levels (D_1_ = control/no drought, D_2_ = drought at tillering stage—DTS, and D_3_ = drought at grain-filling stage—DGS) in wheat showed variability among the treatments and growth-stage-based drought episodes.

The PCA illustrated the component analysis of various wheat parameters in accordance with the contributory fraction of each studied parameter (Figure 8b). Specifically, the coloured scale bars in Figure 8b represented the relative contribution of the growth, yield, morpho-physiological, biochemical, and quality traits of wheat during the application of PGPR and ZnO NPs undergoing drought stress. It was also revealed that the growth stage-based minimum contribution was observed in PPO and POD concentrations (enzymatic antioxidants) among all the 35 studied parameters of wheat under a single use of *Azospirillum brasilense* (T_2_) at drought-stressed grain-filling stage (D_3_ = DGS).

## 4. Discussion

The adverse effects of water deficit situations on wheat growth, development, and physiology are well documented in earlier drought studies [4,6,40,48]. The current field study revealed that PGPR (*Azospirillum brasilense*) and ZnO NPs improved the biological adaptations in wheat undergoing drought. Most recently, Azmat et al. [49] confirmed the restoration of wheat plants’ growth and physiology from drought stress through the synergistic actions of another PGPR (*Pseudomonas* sp.) and ZnO NPs under greenhouse conditions. Saliently, these studies confirmed the synergistic actions of PGPR and ZnO-NPs in improving drought resilience in wheat plants.

Our research showed a significant decrease in per square meter productive tillers and nodes per stem (NS^−1^) under drought as compared to control. Productive tillers and NS^−1^ decreased up to 23% and 21%, and 30% and 10% at tillering (D_2,_ DTS) and GFS (D_3_, DGS), respectively, over respective controls (T_1_). Plant growth reduction had been reported by Raza et al. [50] under water deficit. Likewise, panicle/unit area, grain/panicle, panicle length, PH, etc., in rice and wheat were reported to get significantly affected by the drought [51]. The 10% and 9% reduced inter-nodal length under drought at TS (D_2_, DTS) and GFS (D_3_, DGS) as compared to control (T_1_) at D_1_ (Control) during the current study (Table 1) is similar as described by Boonjung and Fukai [52].

The present field trial at the grain-filling stage (D_3_) exhibited the lowest grains per spike, spikelets, biological, straw plus grain yields, and harvest index under drought as paralleled to control (D_1_ × T_1_). Similarly, 5% plant height and 32% spike length reduction under drought at tillering (T_1_ × D_2_) revealed a significant effect of drought on wheat with variant responses at different growth stages. Wisal et al. [53] reported the same comparable outcomes and proved the positive impact of supplementary irrigation on the restoration of wheat growth for better financial returns. Similarly, Farooq et al. [54] reported a growing-stage-based discrepancy in responses to the drought that may differently distress the final yields.

The use of PGPR and NPs alone or in combination reduced the drought impacts and conserved limited water resources. Their combined efforts proved to be more effective than single use. Similar findings on the sole use of PGPR and ZnO–NPs were stated by Raheem et al. [55] and Munir et al. [56], respectively, while the effect of combination (PGPR and NPs) was found to be in line with Hussain et al. [57], who reported improved plant height, foliar chlorophyll, productive tillers per unit area, lengths of spike, spikelets, and grains per spike, grain + straw yields and harvest index with a combination of PGPR (*Pseudomonas* sp., *Bacillus* sp.) and nano-fertilizer over control. Similarly, Alharby and Ali [58] reported better rice growth, biomass, and chlorophyll by applying Fe NPs with bacteria (*Staphylococcus aureus*) under chromium stress.

Furthermore, Timmusk et al. [59] stated that the interactive behavior of various PGPR (*Bacillus thuringiensis*, *Paenibacillus polymyxa*, *Alcaligenes faecalis*) and TiO_2_ NPs under field conditions revealed the dissolution of minerals to mineral NPs by PGPR (through plausible chelating of organic acids and anions from roots), which are biocompatible to plants and possibly easier to absorb, thereby enhancing plant growth. Improved wheat growth and drought stress resilience through better biomass, photosynthetic pigments, nutrients, soluble sugars, protein, and plant hormones (auxins, abscisic acid) have also been reported under the alone or combined application of PGPR (*Pseudomonas* sp.) and ZnO NPs by Azmat et al. [49]. The highest results under combined treatment (PGPR and ZnO NPs) (T_4_) in the present study may be due to the synergism of PGPR and ZnO NPs. Our data revealed that grain protein contents increased under drought treatments while grain zinc contents and grain weight per spike decreased. The combination of *Azospirillum brasilense* and ZnO NPs (T_4_) improved 7% protein, 17% grain weight per spike, and 54% grain Zn contents over drought even when this combined treatment was applied at a vegetative phase of plant life (D_2_) (Table 3). Sial et al. [60] reported higher protein levels in late-sown wheat, possibly due to low grain weight or heat shock protein production under abiotic stresses. Consequently, Farahbakhsh and Sirjani [61] concluded that Zn and mycorrhizal fungi combination as a combined application to restore wheat yield and its grain quality (grain Zn, protein, gluten, phytic acid, etc.) under pre-harvest drought stress and conserving 450 m^3^ ha^−1^ of water.

The lower values of photosynthetic pigments (chlorophyll a, b and a + b) were found in drought treatments (D_2_, DTS and D_3_, DGS) as compared to non-drought (D_1_-control); conversely, NPs and PGPR increased their values. The lower levels of photosynthetic pigments were attributed to increased ROS activity [16]. Babaei et al. [37] earlier observed that NPs of Zn-Fe oxides with PGPR increased the wheat chlorophyll a, b, a + b under salinity stress. The current study observed declined foliar water relations under the simulated water deficit treatments. Foliar RWC and transpiration showed decreasing trend under drought over control. The lowest and highest leaf water and osmotic potentials were recorded under drought and control, respectively. The wheat and rice plants under water stress had lower leaf water potential and relative water content than well-watered conditions [62]. Nawaz et al. [63] linked reduced water relations with the inception of osmotic stress (secondary stress). After foliar application of a natural plant hormone (zeatin, a cytokinin), Raza et al. [48] reported increased RWC under stimulation of endogenous hormone levels in wheat where the higher RWC can maintain water and osmotic potentials. Raza et al. [64] reported that macromolecules hydrolyzed into minor ones leading to more osmolytes production in tissues and, improving osmotic potential to cope with the partial root drying conditions.

Our data showed higher N (11% and 8%), P (51% and 17%), and K (1.1% and 2%) uptake by wheat aerial organs by T_4_ at TS (D_2_) and GFS (D_3_), respectively, over their respective controls. Similarly, Zn and Fe uptake by wheat also improved when PGPR and NPs applied. Turan et al. [65] reported that *Azospirillum* (sp. 245) delivered higher N, P, K, Fe, Zn, and S content in wheat (leaf, grain, and straw). Improved nutrient uptake in drought-stressed wheat was also observed under the combined application of PGPR and ZnO NPs [49]. Similarly, improved micronutrient bioavailability under the application of Fe NPs and PGPR (*Staphylococcus aureus*) on rice plants under metal stress had been reported by Alharby and Ali [58]

Du et al. [66] reported enhanced Zn accumulations under ZnO NPs alone, which is similar to our findings. Moreover, no risk and specific toxicity had been found with ZnO NPs as zinc remains no longer in the form of zinc oxide within the wheat plant. In wheat, zinc mostly exists in the form of Zn phosphate (non-hazardous) and may coexist with P and S [67,68]. Interestingly, drought tolerance has been confirmed earlier by the combined activities of soil microbes (*Pseudomonas chlororaphis*, *Ensifer meliloti* [synonym, *Rhizobium meliloti*]) and ZnO NPs and plausibly through nutrient mobilization [69,70].

Enhanced proline and total phenolics were observed with T_4_ at 15% and 22%, and 20% and 28%, at TS (D_2_), respectively, and 18% and 2.5%, and 19% and 4%, at GFS (D_3_) as compared to exclusive PGPR (D_2_ × T_2_, D_3_ × T_2_) and ZnO NPs (D_2_ × T_3_, D_3_ × T_3_) respectively. These increases might be ascribed to a natural defense system (NDS) to minimize the toxic ROS [71]. NPs act as nano fertilizers in delivering better nutrients’ bioavailability [67], and Awan et al. [36] linked this improvement to enhanced metabolism and, as a result, greater availability of proline and phenolics and conferring better stress resilience (drought) in wheat under its different growth stages. In addition, PGPR could synergistically enhance these drought tolerance phenomena with their optimized phytohormonal balance [26,29] and nutrient bioavailability [72]. These nanomaterials and microbes’ interactions are plausible explanations for which *Azospirillum brasilense* and ZnO NPs delivered high TP and proline under drought-sensitive stages of our field-grown wheat. Interestingly, similar increased enzymatic and non-enzymatic activities had also been reported in chromium-stressed rice by interactive NPs and bacteria [58]. Zulfiqar et al. [73] also reported nanoparticles-based fertilizers as abiotic stress tolerance enhancers, and their application in combination with compatible microorganisms (e.g., PGPR) provided great additional benefits in the agrifood biotechnology and horticulture sectors by increasing the crop production potentials under the current climate change scenarios (drought, extreme temperature). Thus, nano biofertilizers reduced leaching and volatilization as compared to conventional fertilizers and offering a viable alternative to increase growers’ profit margin through improved yield and product quality.

Our data revealed that drought generally increased the levels of antioxidant enzymes as compared to control in wheat. Such enzymatic changes might be due to ROS overproduction and a heightened antioxidant defense system [48,74]. Accordingly, *A. brasilense* and ZnO NPs (T_4_) further increased wheat antioxidant enzymes under drought: APX at 28% and 14%, CAT at 19% and 35%, POD at 27% and 26%, SOD at 33% and 43%, and PPO at 35% and 37% at TS (D_2_) and GFS (D_3_), respectively, over respective drought controls (T_1_). Yavas and Unay [75] reported significantly increased CAT and SOD activities in wheat crops under drought by Zn application. Similarly, NPs promoted higher levels of APX, POD and PPO and these observations were reported by Iannone et al. [76], Arough et al. [77], and Chiahi et al. [78], respectively. PGPR also increased antioxidant enzymatic activities under drought [79,80]. The combination of PGPR with Zn-Fe oxides NPs proved to be a promoter of CAT, POD, and PPO in wheat under salt stress [37]. Protection of wheat and rice plants from drought and heavy metal stress, respectively, is also evident through the co-application of PGPR and ZnO NPs, and, PGPR and Fe NPs, respectively, by producing more antioxidant enzymes (APX, CAT, SOD, and POD) and proline [49,58].

## 5. Conclusions

Any drought episode would induce numerous morpho-physiological and biochemical changes during various growth stages (tillering, grain filling) to alter plant performance, quality, and yield in wheat. Interestingly, ZnO NPs, PGPR, and their co-application ameliorated the reduction in growth and yield through increased drought tolerance, sustained nutrient uptake, and optimization of water relations, and levels of antioxidants (APX, CAT, POD, PPO, proline, and TP) and photosynthetic pigments (Chl. a + b, b, a and carotenoids). Based on these results, the ZnO NPs and *Azospirillum brasilense* combination was concluded as the effective combination for wheat against drought with improved final harvest and grain quality attributes. This combination may also be considered an effective strategy for better cereal bio-fortification under drought. Moving forward, further field studies under different soil types and cultivation environments are required to validate the combined nanomaterials and microbial approach to improving crop resilience.

## Figures and Tables

**Table 1:** Effects of applying *Azospirillum brasilense* and zinc oxide nanoparticles on wheat resilience to drought at different growth stages.

Experimental Factorization		Growth Promoting Characters Under Field Trial				
Drought Levels	Treatments	PT	Int.L	PH	SL	NSPS
**D_1_ = no drought/control**	T_1_	356.0 ± 2.3 ^c^	9.4 ± 0.1 ^d^	88.2 ± 1.1 ^g^	12.3 ± 1.5 ^c^	20.1 ± 1.0 ^cd^
	T_2_	360.0 ± 1.5 ^ab^	9.6 ± 0.1 ^b^	90.1 ± 0.7 ^c^	13.2 ± 0.6 ^bc^	21.5 ± 0.5 ^ab^
	T_3_	358.0 ± 1.3 ^bc^	9.5 ± 0.1 ^c^	89.1 ± 1.2 ^f^	13.0 ± 2.1 ^bc^	21.0 ± 0.6 ^bc^
	T_4_	362.0 ± 3.5 ^a^	9.7 ± 0.1 ^a^	92.4 ± 2.7 ^a^	14.7 ± 0.6 ^a^	22.5 ± 0.6 ^a^
**D_2_ = DTS**	T_1_	272.0 ± 1.8 ^j^	8.4 ± 2.5 ^j^	83.1 ± 1.8 ^l^	8.2 ± 0.3 ^e^	17.3 ± 2.5 ^g^
	T_2_	292.0 ± 2.0 ^f^	8.6 ± 0.1 ^h^	86.0 ± 1.6 ^j^	9.1 ± 1.0 ^e^	18.3 ± 2.2 ^fg^
	T_3_	285.0 ± 1.4 ^g^	8.3 ± 2.0 ^k^	84.0 ± 1.2 ^k^	8.9 ± 0.19 ^e^	18.1 ± 1.0 ^fg^
	T_4_	314.0 ± 2.0 ^d^	8.7 ± 0.1 ^g^	87.1 ± 1.2 ^i^	10.4 ± 2.2 ^d^	18.7 ± 2.3 ^ef^
**D_3_ = DGS**	T_1_	278.0 ± 2.6 ^i^	8.5 ± 1.4 ^i^	88.0 ± 2.5 ^h^	10.4 ± 3.1 ^d^	15.6 ± 0.6 ^h^
	T_2_	282.0 ± 2.0 ^gh^	8.8 ± 0.1 ^f^	90.0 ± 2.7 ^d^	11.0 ± 7.1 ^d^	18.7 ± 2.1 ^ef^
	T_3_	280.0 ± 1.6 ^hi^	8.8 ± 1.7 ^g^	89.2 ± 2.0 ^e^	10.9 ± 1.0 ^d^	18.0 ± 3.2 ^fg^
	T_4_	306.0 ± 1.0 ^e^	9.2 ± 0.1 ^e^	92.2 ± 3.7 ^b^	13.7 ± 4.0 ^ab^	19.6 ± 1.6 ^de^

Note: Productive tillers (PT, m^−2^), internodal length (Int.L, cm), plant height (PH, cm), spike length (SL, cm), and number of spikelets per spike (NSPS). Data in the table are interactive means ± standard deviation; the means sharing the same letters are not significantly different at 5% probability level (*n* = 3). DTS = drought at tillering, DGS = drought at grain filling, T_1_ = control, T_2_ = single use of *Azospirillum brasilense*, T_3_ = single use of ZnO NPs, and T_4_ = co-application of PGPR and NPs.

**Table 2:** Drought-ameliorating effects of PGPR and ZnO nanoparticles on number of grains (NGS), 1000 grain weight (1000 Gwt. g), grain yield (T ha^−1^), straw yield (SY, T ha^−1^), and harvest index (HI,%) of wheat under different drought levels.

Experimental Factorization		Yield Promoting Characters Under Field Trial				
Drought Levels	Treatments	NGS	1000 Gwt.	GY	SY	HI
**D_1_ = no drought/control**	T_1_	40.1 ± 1.8 ^d^	39.0 ± 1.0 ^cd^	4.6 ± 1.2 ^d^	7.9 ± 0.6 ^d^	36.9 ± 1.5 ^d^
	T_2_	42.0 ± 1.0 ^b^	40.0 ± 0.0 ^ab^	4.9 ± 0.9 ^b^	8.7 ± 0.2 ^b^	36.3 ± 1.0 ^e^
	T_3_	41.0 ± 1.0 ^c^	39.5 ± 0.8 ^bc^	4.7 ± 0.1 ^c^	8.5 ± 2.0 ^c^	35.7 ± 1.1 ^g^
	T_4_	43.0 ± 1.2 ^a^	40.5 ± 2.2 ^a^	5.2 ± 0.1 ^a^	9.3 ± 2.3 ^a^	36.0 ± 1.0 ^fg^
**D_2_ = DTS**	T_1_	37.0 ± 1.7 ^g^	36.1 ± 1.2 ^g^	4.1 ± 0.1 ^h^	5.2 ± 2.0 ^l^	44.6 ± 1.4 ^a^
	T_2_	39.0 ± 1.4 ^e^	38.0 ± 1.0 ^e^	4.3 ± 1.6 ^g^	7.7 ± 1.7 ^f^	36.0 ± 1.0 ^ef^
	T_3_	38.2 ± 2.4 ^f^	37.0 ± 1.0 ^f^	4.4 ± 2.2 ^f^	6.8 ± 1.3 ^j^	39.4 ± 1.1 ^b^
	T_4_	41.0 ± 0.8 ^cd^	38.5 ± 0.1 ^de^	4.6 ± 1.2 ^e^	7.7 ± 0.6 ^e^	37.3 ± 1.0 ^c^
**D_3_ = DGS**	T_1_	32.0 ± 2.0 ^i^	23.6 ± 2.5 ^k^	2.6 ± 0.9 ^l^	6.0 ± 1.2 ^k^	30.3 ± 1.3 ^i^
	T_2_	35.4 ± 1.3 ^h^	27.8 ± 1.7 ^i^	3.0 ± 0.1 ^j^	7.2 ± 1.0 ^h^	29.0 ± 2.1 ^j^
	T_3_	35.0 ± 0.9 ^h^	26.2 ± 0.3 ^j^	2.8 ± 1.0 ^k^	7.1 ± 0.3 ^i^	28.7 ± 1.2 ^k^
	T_4_	38.7 ± 1.1 ^ef^	29.8 ± 1.0 ^h^	3.6 ± 1.2 ^i^	7.4 ± 2.1 ^g^	28.7 ± 1.1 ^k^

Data in the table are interactive means ± standard deviation; the means sharing different letters are significantly different at 5% probability level (*n* = 3). DTS = drought at tillering, DGS = drought at grain filling, T_1_ = control, T_2_ = single use of *Azospirillum brasilense*, T_3_ = single use of ZnO NPs, and T_4_ = co-application of PGPR and NPs.

**Table 3:** Responses of wheat to sole or co-application of *Azospirillum brasilense* and Zinc oxide nanoparticles (ZnO NPs) during drought.

Experimental Factorization		Morphological and Grain Quality Traits Under Field Trial				
Drought Levels	Treatments	NS^−1^	NFT	SpWt.	Gprot.	Gzn
**D_1_ = no drought/control**	T_1_	3.3 ± 0.1 ^cd^	10.0 ± 1.3 ^de^	2.0 ± 0.1 ^d^	9.3 ± 0.1 ^l^	12.0 ± 0.1 ^i^
	T_2_	4.0 ± 0.1 ^ab^	7.0 ± 2.1 ^fg^	2.1 ± 1.0 ^b^	11.2 ± 0.1 ^e^	16.7 ± 0.1 ^d^
	T_3_	3.9 ± 0.2 ^ab^	8.0 ± 1.2 ^ef^	2.1 ± 0.2 ^c^	10.3 ± 0.8 ^j^	14.5 ± 0.1 ^e^
	T_4_	4.3 ± 0.1 ^a^	5.0 ± 1.2 ^g^	2.3 ± 0.1 ^a^	12.2 ± 0.1 ^a^	18.3 ± 0.2 ^b^
**D_2_ = DTS**	T_1_	2.3 ± 0.9 ^f^	22.0 ± 0.9 ^a^	1.5 ± 0.1 ^h^	10.0 ± 0.1 ^k^	11.0 ± 0.1 ^k^
	T_2_	2.6 ± 0.3 ^ef^	14.0 ± 2.2 ^bc^	1.7 ± 0.1 ^f^	10.8 ± 0.1 ^h^	12.4 ± 0.2 ^h^
	T_3_	2.6 ± 0.1 ^ef^	16.0 ± 2.0 ^b^	1.6 ± 1.4 ^g^	10.7 ± 0.3 ^i^	11.6 ± 0.9 ^j^
	T_4_	3.3 ± 0.1 ^cd^	12.0 ± 1.3 ^cd^	1.8 ± 0.2 ^e^	10.8 ± 0.1 ^g^	16.9 ± 0.1 ^c^
**D_3_ = DGS**	T_1_	3.0 ± 1.2 ^de^	11.0 ± 1.4 ^d^	1.0 ± 0.1 ^l^	11.0 ± 0.5 ^f^	9.0 ± 1.1 ^l^
	T_2_	3.8 ± 0.1 ^abc^	12.0 ± 1.2 ^cd^	1.1 ± 0.1 ^j^	11.9 ± 0.1 ^c^	13.6 ± 0.2 ^g^
	T_3_	3.6 ± 0.1 ^bc^	11.0 ± 1.3 ^d^	1.0 ± 0.1 ^k^	11.4 ± 0.1 ^d^	14.3 ± 0.9 ^f^
	T_4_	4.0 ± 0.1 ^ab^	11.0 ± 1.7 ^d^	1.2 ± 0.1 ^i^	12.1 ± 0.2 ^b^	19.5 ± 0.1 ^a^

Note: Number of nodes per stem (NS^−1^), non-fertile tillers (NFT, m^−2^), grain weight per spike (SpWt. g), grain protein (Gprot.%) and grain zinc contents (Gzn, mg kg^−1^). Data in the table are interactive means ± standard deviation; the means sharing dissimilar letters are significantly different at 5% probability level (*n* = 3). DTS = drought at tillering, DGS = drought at grain filling, T_1_ = control, T_2_ = single use of *Azospirillum brasilense*, T_3_ = single use of ZnO NPs, and T_4_ = co-application of PGPR and NPs.

## Data Availability

The data obtained in this study are presented “as is” on at least one of the figures or tables embedded in the manuscript.
